# Hypothalamic endocannabinoids in obesity: an old story with new challenges

**DOI:** 10.1007/s00018-021-04002-6

**Published:** 2021-10-31

**Authors:** Cristina Miralpeix, Ana Cristina Reguera, Anna Fosch, Sebastian Zagmutt, Núria Casals, Daniela Cota, Rosalía Rodríguez-Rodríguez

**Affiliations:** 1 University of Bordeaux, INSERM, Neurocentre Magendie, U1215, 3300 Bordeaux, France; 2 Basic Sciences Department, Faculty of Medicine and Health Sciences, Universitat Internacional de Catalunya, Josep Trueta S/N, 08195 Sant Cugat del Vallès, Spain; 3 Centro de Investigación Biomédica en Red de Fisiopatología de La Obesidad Y La Nutrición (CIBEROBN), Instituto de Salud Carlos III, 28029 Madrid, Spain

**Keywords:** Endocannabinoid, Cannabinoid receptors, Hypothalamus, Obesity, Energy balance

## Abstract

The crucial role of the hypothalamus in the pathogenesis of obesity is widely recognized, while the precise molecular and cellular mechanisms involved are the focus of intense research. A disrupted endocannabinoid system, which critically modulates feeding and metabolic functions, through central and peripheral mechanisms, is a landmark indicator of obesity, as corroborated by investigations centered on the cannabinoid receptor CB1, considered to offer promise in terms of pharmacologically targeted treatment for obesity. In recent years, novel insights have been obtained, not only into relation to the mode of action of CB receptors, but also CB ligands, non-CB receptors, and metabolizing enzymes considered to be part of the endocannabinoid system (particularly the hypothalamus). The outcome has been a substantial expansion in knowledge of this complex signaling system and in drug development. Here we review recent literature, providing further evidence on the role of hypothalamic endocannabinoids in regulating energy balance and the implication for the pathophysiology of obesity. We discuss how these lipids are dynamically regulated in obesity onset, by diet and metabolic hormones in specific hypothalamic neurons, the impact of gender, and the role of endocannabinoid metabolizing enzymes as promising targets for tackling obesity and related diseases.

## Introduction

Obesity is a global epidemic and a major risk factor for type-2 diabetes, cardiovascular diseases, cancer, and more recently, for COVID-19 outcomes [1, 2]. If the global trend continues, worldwide obesity prevalence in 2025 will be around 21% for women and 18% for men, representing a serious burden to public health, and having major economic and social consequences [3]. Although lifestyle modifications (caloric restriction and exercise) are an integral part of obesity management, they are often insufficient on their own. In recent decades, while anti-obesity drugs have become available into the market, none are able to ameliorate obesity over the long term. This is evidence that highlights the complexity of the molecular mechanisms underlying obesity development and progression, hindering the development of efficacious drugs. There is an urgent need to understand the pathophysiology of obesity, so as to develop long-term clinically efficacious therapeutic strategies with minimal side effects.

Obesity arises from a dysregulation in energy metabolism, in a process finely controlled by the central nervous system (CNS), and particularly by the hypothalamus, which has emerged as a master regulator of whole-body energy homeostasis [4, 5]. Hormone- and nutrient-sensing hypothalamic nuclei orchestrate central and peripheral responses for maintaining normal body weight, food intake, energy expenditure and nutrient partitioning. In those nuclei, specialized neuronal populations are inter-connected transmitting and receiving information from various extra-hypothalamic brain regions in order to coordinate whole-body energy balance [5, 6]. Evidence also suggests the key participation of non-neuronal cells, such as microglia and astrocytes, whose diet-dependent changes lead to insulin resistance and obesity [7, 8].

A landmark indicator of obesity is dysregulation of the endocannabinoid system (ECS), recognized to play a critical role in regulating energy balance through central and peripheral mechanisms [9, 10]. In modern societies where overeating is normal, the ECS ends up favoring obesity [11], as solidly corroborated by investigations using models of mice lacking cannabinoid (CB) receptors that develop alterations in body weight, fat mass, and food intake [12, 13]. Accordingly, cannabinoid type 1 (CB1) receptor inhibition by synthetic drugs such as rimonabant (CB1 inverse agonist) have been shown to produce promising anti-obesity effects in both animal models and obese patients [9]. Unfortunately, the neuropsychiatric side effects reported for some patients led to rimonabant’s withdrawal from the European market in 2009 [14]. It is clear that further knowledge on the mode of action of the ECS, mostly in the CNS, is required to shed light into its pathophysiological functions in humans and to be able to target the ECS in a more selective and specific manner [14, 15].

In recent years, several studies have provided novel insights to the ECS, not only in relation to the mode of action of CB receptors, but also CB ligands, non-CB receptors and metabolizing enzymes part of the ECS, particularly in the hypothalamus; this has substantially expanded knowledge of this complex signaling system and potentially facilitates drug development. The aim of this review was to update and discuss recent findings and provide an up-to-date overview of the role of the hypothalamic ECS in driving obesity. In particular, here we pay special attention to novel aspects including: (i) endocannabinoid dynamic changes and levels in the hypothalamus as biomarkers of obesity progression; (ii) recent findings on CB1 in specific cell types, as hypothalamic neurons and astrocytes, or even in intracellular compartments such as membrane associated with mitochondria, and the possible impact on energy balance; and (iii) other new insights going beyond cannabinoids, such as manipulation of proteins involved in endocannabinoids metabolism in the hypothalamus as promising targets against obesity.

## Old and new components of the endocannabinoid system

**Fig. 1:**
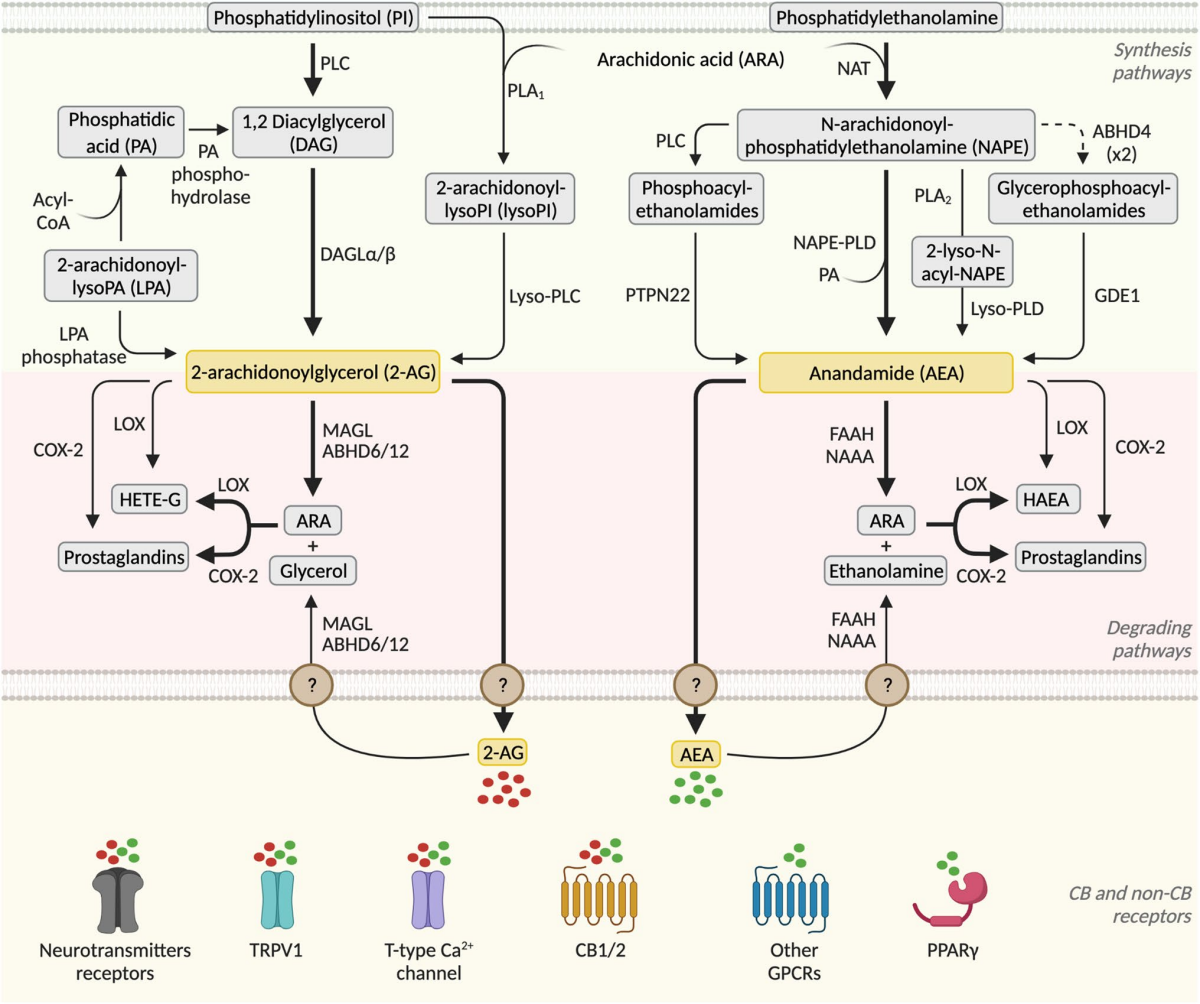
Schematic representation of endocannabinoids synthesis and degradation pathways with special attention to ECS enzymes *(Figure created with BioRender.com)*

The ECS is a complex and well-conserved system [16], which consists of the CB receptors, their endogenous ligands called endocannabinoids and their synthesis and degradation pathways (Fig. 1). Concerning the CB receptors, two have been identified, namely CB1 [17] and cannabinoid type 2 (CB2) [18], both belonging to the G protein-coupled receptor (GPCR) family. The endocannabinoids are n-6 polyunsaturated fatty acids (PUFA) derived from membrane phospholipids precursors and arachidonic acid (ARA); the best characterized ones are 2-arachydonoyl glycerol (2-AG) and arachidonoylethanolamide or anandamide (AEA). Despite some controversies [19], it is generally accepted that, within the CNS and on neuronal activation, endocannabinoids are synthesized on demand at the postsynaptic terminals from membrane lipid precursors. The endocannabinoids then retrogradely act on CB1 receptors located in the presynaptic site, where they regulate ion channel activity and inhibit neurotransmitter release. In this section, we will introduce well-described components of the ECS, paying special attention to the endocannabinoids metabolizing enzymes and to novel receptors and proteins recently suggested to be part of the ECS.

### Cannabinoid and non-cannabinoid receptors

The CB1 receptor is probably one of the most highly expressed GPCRs in the brain [20]. It is present in different brain areas, including those associated with the regulation of feeding behavior and whole body energy balance, such as the hypothalamus, the brainstem, and the corticolimbic system [21, 22]. Within different neuronal populations, the CB1 receptor is mainly present not only in GABAergic and glutamatergic neurons, but also expressed in serotonergic, noradrenergic, and cholinergic neurons [20]. It has recently been found that, besides its classical localization in presynaptic membranes, the CB1 receptor is also associated with the mitochondria membranes (mtCB1) of neurons, where it regulates cellular respiration, energy production, and mitochondrial motility [23, 24]. Interestingly, it has been suggested that mtCB1 receptors in pro-opiomelanocortin (POMC) neurons (a hypothalamic neuronal population critically controlling energy balance) mediate cannabinoid-induced food intake [25]. In addition to neurons, CB1 and mtCB1 receptors are also expressed in astrocytes, where they regulate neuroinflammation, neurotransmission, and glucose metabolism [26–28]. Since CB1 receptors are structurally associated with astrocytes that form the tripartite synapse [29], and glia cells are involved in the control of food intake and metabolism, in part by regulating neuronal circuits [30], it would be highly interesting to study the role of the CB1 receptor in hypothalamic astrocytes in obesity.

CB1 control of energy metabolism is not restricted to the CNS, since this receptor is also expressed in peripheral organs such as the adipose tissue, liver, skeletal muscle, pancreas, kidney, and gastrointestinal tract [14]. Regarding the CB2 receptor, this was initially categorized as the peripheral CB receptor since it is expressed in the adipose tissue, muscle, liver, spleen, testes, and immune system cells [31]. However, it is also present in neurons and astrocytes [32, 33], although to a lesser extent than the CB1 receptor, and especially in microglia [31]. The CB2 receptor exerts important neuroprotective and anti-inflammatory actions and so has become a potential target for neurodegenerative diseases [31]. In obesity, it has been shown that CB2 plays a protective role against this disease, thanks to its anti-inflammatory function [34–36]; however, its role has been little studied [37] and results are controversial, highlighting the need for more research on the potential role of CB2 in obesity.

Classically, CB1 and CB2 receptors are coupled to G_i/o_ proteins and their signal transduction is mediated by inhibition of adenylyl cyclase (AC) and voltage-gated calcium channels as well as by activation of MAPK and potassium channels [20]. However, AEA and synthetic cannabinoids are able to exert non-CB1 and CB2 mediated effects in CB1-KO mice [38, 39], suggesting the existence of alternative molecular targets (Fig. 1). For instance, endocannabinoids activate transient receptor potential cation channels; the most studied of these channels is vanilloid receptor type 1 (TRPV1), typically activated by temperature and regulating nociception [40]. Endocannabinoids have also been proposed as ligands of orphan GPCR such as GPR55, GPR110, and GPR119. Nevertheless, it is still a matter of debate whether these receptors physiologically signal endocannabinoid-related responses [41]. In addition, some studies have suggested that endocannabinoids can bind to neurotransmitter receptors such as those for serotonin, glycine, and GABA_A_, as well as to peroxisome proliferator-activated receptors (PPAR) [42]. Since endocannabinoids are widely synthetized in the CNS, to fully understand their action it is important to consider the effect mediated by those non-CB receptors.

### The endocannabinoids and their synthesis and degradation pathways

AEA and 2-AG are the two best-characterized endocannabinoids to date. Both endocannabinoids, highly lipophilic ligands derived from both membrane phospholipids and ARA, belong to the large family of *N*-acylethanolamines (NAE), in the case of AEA, and monoacylglycerols (MAG) in the case of 2-AG. Although the two lipids are structurally similar, they present differences in their receptor affinity, concentrations, and metabolic enzymes, suggesting different roles [43]. Indeed, AEA acts as a partial agonist of the CB1 receptor and as a weak partial agonist/antagonist of the CB2 receptor whereas 2-AG is a full agonist of both CB receptors [42]. Given that 2-AG levels in the brain are ~ 170 times higher than those of AEA [44], 2-AG has been proposed as the main endogenous agonist for both CB receptors [42], and AEA as an ubiquitous ligand also able to act on other non-CB receptors, as discussed previously.

Within the brain, endocannabinoid levels are finely regulated by their enzymes of synthesis and degradation, both in presynaptic and postsynaptic sites, forming a redundant system involving several pathways [45] (Fig. 1). The major pathway of AEA synthesis starts with phosphatidylethanolamine and ARA being transformed into *N*-arachidonoylphosphatidylethanolamine (NAPE) by the enzyme *N*-acyltransferase (NAT). NAPE is then hydrolyzed by the NAPE selective phospholipase D (NAPE–PLD), producing AEA and phosphatidic acid (Fig. 1). Although the NAPE-PLD pathway is the best characterized, there are three additional pathways, involving the serine α/β-hydrolase domain containing 4 (ABHD4) and glycerophosphodiesterase 1 (GDE1), the protein tyrosine phosphatase PTPN22, and lyso-NAPE [45]. Regarding 2-AG production, the precursor phosphatidylinositol (PI) is converted into 1,2 diacylglycerol (1,2-DAG) by phospholipase C (PLC), and then into 2-AG by diacylglycerol lipase (DAGL). An alternative pathway for 2-AG synthesis is when PI is transformed into 2-arachidonoyl-lysophospholipid (lyso-PI) by the phospholipase A1 (PLA_1_), finally generating 2-AG (Fig. 1) [45].

In addition to the synthesizing machinery, endocannabinoids are tightly regulated by their degradation enzymes (Fig. 1). Following reuptake of endocannabinoids after CB receptors activation, AEA is cleaved principally by the fatty acid amide hydrolase (FAAH) into ethanolamine and ARA. *N*-acylethanolamine-hydrolyzing acid amidase (NAAA) has been also described as a degradation enzyme of the *N*-acylethanolamines [46]. 2-AG is mainly inactivated by monoacylglycerol lipase (MAGL) and, secondarily, by ABHD6 and ABHD12 [47] into glycerol and ARA.

Endocannabinoids are also substrates for enzymes that oxygenate ARA, such as cyclooxygenase-2 (COX-2), the lipoxygenases (LOX), and cytochromes, representing a link between the endocannabinoids and the eicosanoid systems [48] (Fig. 1). These inactivating pathways generate AEA and 2-AG hydroxy-derivatives (HAEA and HETE-G, respectively), as well as prostaglandin ethanolamides or prostamides and prostaglandin glycerol esters [48]. However, the effect of these derivatives in metabolism remains to be clarified. Although the endocannabinoid synthesis and degradation pathways might seem redundant, they are multiple targets for the development of pharmacological treatments that aim to reduce or enhance endocannabinoid levels [49].

Besides 2-AG and AEA, there are secondary endocannabinoid derivatives from the n-6 PUFA ARA, such as virodhamine and *N*-arachidonoyl dopamine [45, 50], as well as other n-3 PUFA derivatives of docosahexaenoic acids (DHA) and eicosapentaenoic acid (EPA) [45]. The balance between n-3 and n-6 PUFA precursors obtained from the diet can enhance or reduce endocannabinoid levels in a time- and tissue-specific manner [51]. Consequently, the control of endocannabinoid precursors in the diet may represent a potential therapeutic strategy against obesity (further discussed in Sect. 3.1.2).

After their classic retrograde action on presynaptic CB1 receptors, endocannabinoids are metabolized within the cells; however, the mechanisms underlying uptake and intracellular transport remain unclear. Most studies have focused on AEA transport, since 2-AG is rapidly degraded by multiple enzymes. Initially proposed was a mechanism based on a transmembrane transporter that has not yet been characterized, however. Although other mechanisms have been suggested, such as passive diffusion through the membrane, caveolae-dependent endocytosis, or passive diffusion following carrier-mediated intracellular transport, the exact mechanism leading to AEA and 2-AG transport in the neuronal synapsis remains unknown. Regarding endocannabinoids intracellular trafficking, to date the following four cytosolic lipid-binding proteins have been identified: fatty acid-binding proteins (FABP), sterol carrier protein 2 (SCP-2), heat shock 70 kDa protein (HSP70), and albumin [52–54].

## Hypothalamic endocannabinoid signaling in obesity

The hypothalamus integrates peripheral inputs on energy intake and storage to orchestrate energy balance. Hormones such as leptin, insulin, and ghrelin represent the main energy-related signals that reach the hypothalamus. Lipid-derived signals, such as the endocannabinoids, are also involved in hypothalamic regulation of food intake and whole-body energy metabolism, and, importantly, are modulated by hormones and diet. In this section, we will discuss the impact of altering hypothalamic ECS players (cannabinoid receptors, ligands, and metabolic enzymes) on obesity and the main stimuli driving these changes (diet composition and hormones).

### What modulates hypothalamic endocannabinoids in obesity?

Endocannabinoid levels in different tissues and plasma have been proposed as a potential biomarker of obesity and related diseases [15, 55]. In the hypothalamus, strong evidence suggests a role for 2-AG and AEA changes in the regulation of food intake, food preferences, and body weight control. Variations in hypothalamic 2-AG and AEA levels strictly depend on metabolic hormones, diet composition and nutritional state, and interestingly, these responses are sexually dimorphic.

#### Metabolic hormones

Fluctuations of endocannabinoid levels in the hypothalamus in relation to nutritional status have been linked to the action of metabolic hormones involved in regulating food intake, peripheral metabolism, and energy expenditure. The most studied hormone to date is leptin, an appetite suppressant produced by adipocytes to signal energy surplus, thereby reducing food intake and inducing energy expenditure [56]. In 2001, Di Marzo et al. [57] were the first to relate leptin to the ECS, demonstrating that defective leptin signaling, observed in genetic models of obesity such as obese Zucker rats, and *db/db* and *ob/ob* mice, was associated with elevated hypothalamic endocannabinoids, especially 2-AG [57] (Table 1). Moreover, acute intravenous administration of this hormone restored hypothalamic 2-AG and AEA in *ob/ob* mice, which genetically lack leptin. In line with those results, Balsevich et al. [58] also observed an increase in hypothalamic endocannabinoids in *ob/ob* mice (Table 1). Therefore, increased endocannabinoid levels in the hypothalamus in genetically obese animals would suggest that these mediators contribute to hyperphagia and obesity due to lack of leptin or of its action.

**Table 1.** 2-AG and AEA level fluctuations in hypothalamus or whole brain

Experimental approach	Species	Experimental model	Whole brain		Hypothalamus		Refs.
			2-AG	AEA	2-AG	AEA	
Hormones							
Leptin	Rat	Lean rat (ICV)	–	–	↓	↓	[57]
	Mouse	*Ob/ob* mice (IV)	ns	ns	↑	↑	[57]
	Mouse	Lean mice (IP)	–	–	ns	↓	[58]
	Mouse	Obese DIO mice (IP)	–	–	ns	ns	[58]
	Mouse	Lean mice (ICV)	–	–	↑	↑	[59]
Ghrelin	Mice	IP (1 h)	–	–	↑	↑	[75]
Insulin	Rat	Fasting (overnight), intra-mediobasal hypothalamus infusion (2 h)	–	–	ns (a)	ns (a)	[73]
Dietary-based approach							
Fasting	Rat	Food deprivation for 24 h	–	–	↑	ns	[80]
	Mouse	Food deprivation for 24 h	↑	ns	–	–	[83]
	Mouse	Food deprivation for 24 h	–	–	ns	↑	[84]
Diet restriction	Mouse	Food restriction diet for 12 days. Restricted diets to 60% (57 kcal/week), 50% (47.5 kcal/week) or 40% (38 kcal/week) compared non-restricted diet (95 kcal/week) Note: 2-AG reduction also observed in hippocampus, but the effect was independent on the % of caloric restriction	–	–	↓	–	[83]
HFD	Mouse	ICR mice. 60% kcal from fat for 3–7 days Note: Pre-conditioning test results	–	–	ns	–	[85]
	Mouse	ICR mice. 60% kcal from fat for 14–42 days Note: Pre-conditioning test results	–	–	↑	–	[85]
	Mouse	C57BL/6 N. 45% kcal from fat for 10 days Note: Results after a conditioning test for 10 days	–	–	↑	–	[87]
	Mouse	C57BL/6 J. 60% kcal from fat for 7–28 days	–	–	↑	↑	[59]
	Mouse	C57BL/6 J. 60% kcal from fat for 60 days	–	–	↓	↓	[59]
	Mouse	C57BL/6 J. 60% kcal from fat for 90 days	–	–	↓	ns	[59]
	Mouse	C57BL/6 J. 49% kcal from fat for 16 weeks	–	–	↑ (b)	ns	[88]
	Mouse	C57BL/6 J. 60% kcal from fat for 19 weeks	–	–	ns	ns	[58]
	Mouse	C57BL/6 J. 60% kcal from fat enriched in linoleic acid for 14 weeks	↑	–	–	–	[92]
	Rat	Wistar rat. 60% kcal from fat for 24 weeks	–	–	↑	ns	[89]
Genetic model of obesity							
	Mouse	*ob/ob*	–	–	ns	↑	[57]
	Mouse	*ob/ob*	–	–	↑ (b)	ns	[88]
	Mouse	*ob/ob*	–	–	↑	↑	[58]
	Mouse	*db/db*	–	–	↑	↑	[57]
	Rat	Zucker-fatty	–	–	↑	ns	[57]
Drugs							
DAGL inhibitor	Mouse	IP administration in mice fed a HFD	–	–	↓	–	[87]

*HFD* high fat diet, *ICV* intracerebroventricular, *IP* intraperitoneal, *IV* intravenous, *LH* lateral hypothalamus, *ns* no significant changes appreciated. A dash (–) means no data available

(a) Levels measured in the region of the mediobasal hypothalamus

(b) Levels measured in the region of the lateral hypothalamus (LH)

Although it is clear that hypothalamic endocannabinoids are under the control of leptin in obese rodents, data for normal weight mice are more complex. On the one hand, acute intravenous administration (30 min) of leptin elicited either a decrease in both 2-AG and AEA levels [57] or a decrease in AEA only, with no changes in 2-AG [58] (Table 1). On the other hand, intracerebroventricular (icv) administration (4 h) of leptin in C57BL/6 lean mice induced a significant increase in both 2-AG and AEA in the hypothalamus, an effect that has been suggested to be due to activation of peripheral thermogenesis rather than to direct leptin action on the hypothalamus [59] (Table 1). Interestingly, those responses were not observed in extrahypothalamic areas, suggesting specific action at the level of the hypothalamus [58, 59].

More intriguingly, the appetite-suppressant action of leptin is CB1-dependent, as a 60% reduction in CB1 receptor expression in the hypothalamus was shown to be sufficient to blunt leptin’s effect on food intake [60]. Different studies have attempted to describe the molecular mechanism linking leptin with the regulation of endocannabinoid levels. The enzyme FAAH has been described to be stimulated by leptin, which would explain the decreased levels of hypothalamic AEA after leptin administration [58].

**Fig. 2:**
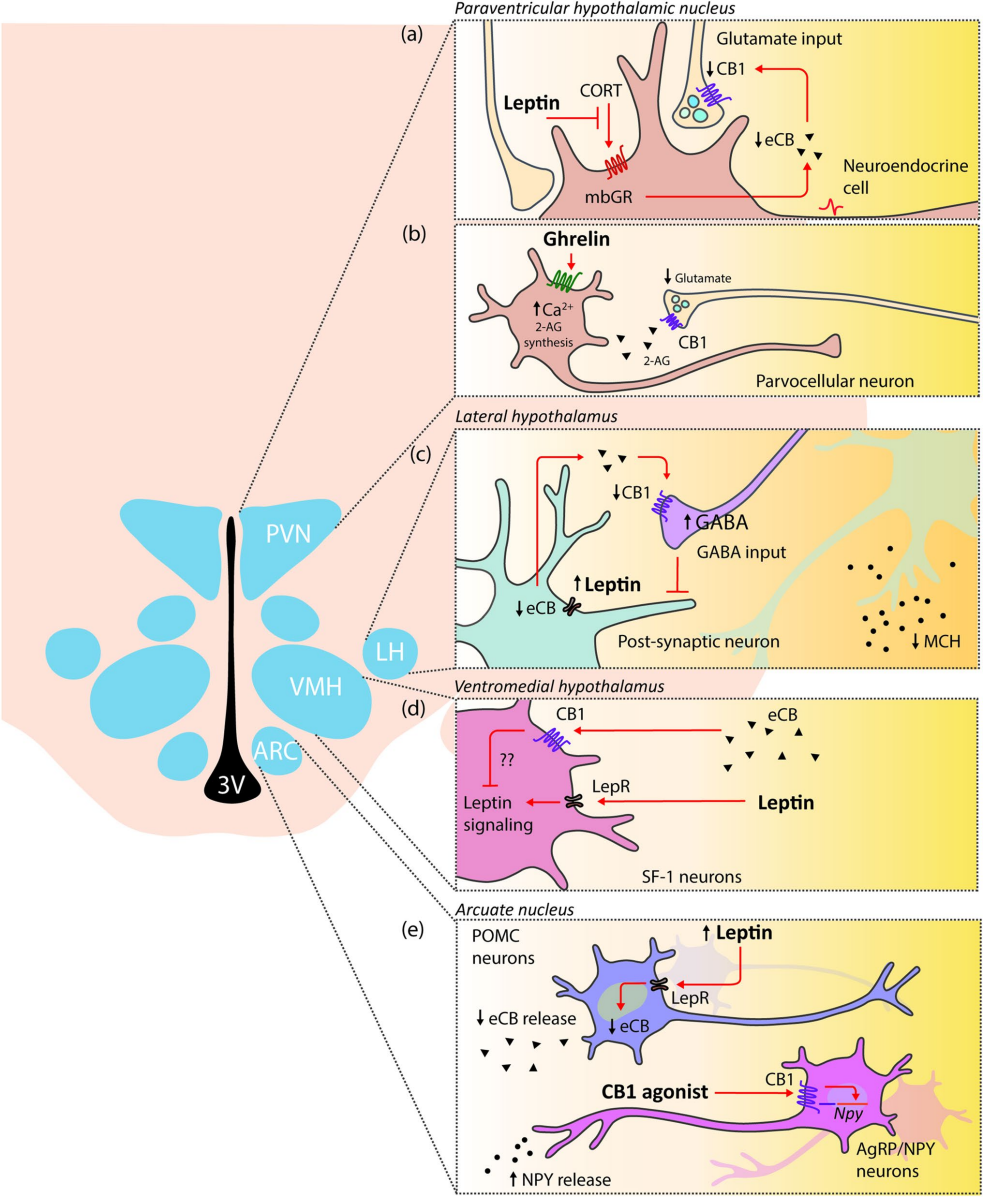
Interactions of leptin and ghrelin with the endocannabinoid system (ECS) in hypothalamic neurons. Peripheral-derived hormones such as leptin and ghrelin stimulate specific areas of the hypothalamus that coordinate the energy balance. The ECS is emerging as an important integrator in these signaling pathway. (a) Leptin acts in parvocellular neuroendocrine cells of the paraventricular nucleus of the hypothalamus (PVN) by reducing the endocannabinoid (eCB) synthesis and release through the membranous glucocorticoid receptor (mbGR) blockade. This effect increases glutamate release from glutamatergic synapsis to the PVN neurons. (b) In addition to leptin, ghrelin acts on parvocellular neurons of the PVN. The bind of ghrelin to its receptor triggers an intracellular Ca^2+^ level increase leading to 2-AG synthesis. The release of 2-AG into the extracellular space, activates the presynaptic CB1 inhibiting the release of glutamate from the axons innervating PVN neurons. (c) In lateral hypothalamus (LH), the activation of leptin receptor (LepR) in prefornical LH neurons results in less synthesis and release of eCB. This effect led to an increase in the GABAergic inhibitory tone to melanocortin-concentrating hormone (MCH) neurons to control food intake and appetite. (d) Leptin also acts on SF-1 neurons of the ventromedial nucleus of the hypothalamus (VMH). Although molecular mechanisms are not fully described, CB-1 dependent endocannabinoid signaling interferes in the leptin activation of SF-1 neurons. (e) Within the arcuate nucleus (ARC), on the one hand leptin activates POMC neurons and reduces eCB synthesis and release. On the other hand, CB1 agonists increase the secretion of NPY in AgRP/NPY neurons

The interplay between leptin and endocannabinoids has been also described for different neuronal populations in the hypothalamus (Fig. 2). Activation of leptin receptor in prefornical neurons of the lateral hypothalamus (LH) drives a reduction in synthesis and release of endocannabinoids, which, in turn, increase the GABAergic presynaptic inhibitory tone to melanocortin-concentrating hormone (MCH) neurons to control food intake [61] (Fig. 2). In line with this evidence, leptin acts in the paraventricular nucleus of the hypothalamus (PVN) parvocellular neuroendocrine cells by reducing endocannabinoid synthesis and release through the glucocorticoid receptor blockade [62] (Fig. 2). This effect increases glutamate release from glutamatergic synapsis to the PVN neurons.

Within the arcuate nucleus (ARC), leptin activates POMC neurons, which classically mediate satiety by engaging the melanocortin system [63, 64]. Under basal conditions, these neurons release endocannabinoids, but after feeding, endocannabinoid release is reduced. Moreover, CB1 agonists in the ARC increase secretion of the appetite-stimulant neuropeptide Y (NPY) [65] from neurons expressing NPY and agouti-related protein (AgRP). NPY/AgRP neurons inhibit the activity of POMC cells, in turn inhibited by leptin [63] (Fig. 2). In addition to neurons of the ARC, leptin also acts on steroidogenic factor 1 (SF1) neurons of the ventromedial nucleus of the hypothalamus (VMH). Interestingly, CB1-dependent endocannabinoid signaling in SF1 neurons interferes with the action of leptin in lean animals, while this same signaling may protect from diet-induced obesity [66] (Fig. 2).

Apart from hypothalamic neurons, it is important to also to mention that there is a leptin-endocannabinoid interplay in astrocytes, as these cells express both CB and leptin receptors. Primary cultures of hypothalamic astrocytes of CB1-KO mice show a reduced expression of the leptin receptor, resulting in weak intracellular glycogen accumulation [67]. Astrocytes offer protection from hypoglycaemia to neurons through their intracellular glycogen [68]; therefore, defective signaling of leptin or endocannabinoids compromises energy availability for neurons.

In addition to leptin, insulin is another appetite-suppressant hormone that has been described to interact with the ECS. Although the interaction between endocannabinoids, insulin, and the regulation of glucose homeostasis has been mainly described for peripheral tissues (reviewed in [14, 69–71]), there is some evidence to suggest that central modulation of CB1 has an impact on insulin effects in peripheral organs. Central activation of the CB1 receptor by the CB1 agonist WIN55,212-2 or the specific agonist arachidonoyl-2’-chloroethylamide (ACEA) acutely impaired insulin action in both liver and adipose tissue [72]. In particular, this central activation of the ECS disrupted the ability of systemic insulin to suppress white adipose tissue (WAT) lipolysis and drove hepatic insulin resistance; this highlights the important role played by the ECS in the brain on the peripheral control of systemic glucose and lipid homeostasis [72]. In line with this, mice lacking CB1 in SF1 neurons of the VMH have increased insulin sensitivity when fed chow diet [66]. This effect, however, may be a consequence of either the impact of the central CB1 receptor on peripheral regulation of insulin response or of reduced fat mass. The hypothesis that central ECS modulates peripheral insulin action but that there is not direct interaction between the ECS and insulin signaling pathway in the CNS is further supported by the lack of change in endocannabinoid levels in the mediobasal hypothalamus of rats after 2 h infusion of insulin in this region [73] (Table 1).

Ghrelin, an orexigenic gut-derived peptide, has also been reported to interact with the ECS. Activation of the growth hormone secretagogue receptor (GHS-R) by ghrelin induces increased appetite and body weight as well as a stimulatory effect on adipose tissue deposition [74]. Interestingly, ghrelin needs an intact and functional ECS to induce its orexigenic effect [75]. Intraperitoneal ghrelin administration raises 2-AG hypothalamic levels in control mice, stimulating food intake, but these effects are not observed in CB1-KO mice [75] (Table 1). Both endocannabinoids and ghrelin activate the hypothalamic AMP-activated protein kinase (AMPK), an important nutritional sensor involved in controlling energy balance [76]. The ability of ghrelin to stimulate hypothalamic AMPK is blunted in CB1-KO mice and also after CB1 pharmacological blockade by intraperitoneal injection of rimonabant [75]. Ghrelin is involved in synaptic excitatory transmission to the parvocellular neurons of the PVN in a CB1-dependent manner (Fig. 2). In binding its receptor to the parvocellular neurons, ghrelin increases intracellular Ca^2+^ levels, leading to 2-AG synthesis. 2-AG is released in the extracellular space where it activates presynaptic CB1, hence inhibiting excitatory input to the parvocellular neurons [75]. An intact endocannabinoid signaling pathway is necessary for ghrelin’s stimulatory effects on hypothalamic AMPK activity and food intake, and for ghrelin’s inhibitory effect on paraventricular neurons. The importance ECS-ghrelin interaction in the PVN has also been demonstrated by Tucci et al. [77], who showed that ghrelin-induced hyperphagia was blocked when ghrelin was administered in the PVN in combination with a sub-anorectic dose of a CB1 antagonist. The interaction between ghrelin and the ECS is bi-directional, since endocannabinoids in turn promote ghrelin synthesis in the gut [78]. Furthermore, intact central and peripheral ghrelin signaling, especially GHS–R1α, is necessary to observe the orexigenic effects of the endocannabinoids [79].

#### Nutritional status, diet-induced obesity, and diet composition

The ability of endocannabinoids to stimulate food intake has been classically associated with activation of reward circuits that promote feeding and increase the incentive value of food [80]. The first direct link between brain endocannabinoid levels and nutritional status was described by Kirkham et al. [80] (Table 1). In that study, in rats, hypothalamic 2-AG increased in response to fasting, but fell after feeding of a palatable food, and remained unchanged in satiated animals. Interestingly, 2-AG was much more sensitive than AEA to nutritional status. Fasting-induced increases in 2-AG were observed in feeding-associated brain regions (limbic forebrain and hypothalamus), but were undetected in brain regions, such as the cerebellum, not directly involved in food intake control. Leaving aside the hypothalamic circuits, the ability of endocannabinoids to stimulate food intake has also been associated with activation of reward circuits, such as those of the nucleus accumbens (NAc), promoting feeding and the incentive value of food [81]. In particular, it has been shown that direct infusion of 2-AG in the NAc induced feeding in satiated rats, in a dose dependent manner [80]. Those results suggest that fluctuations in hypothalamic 2-AG levels, during acute nutritional deficiency, support the behavioral actions of endocannabinoids (i.e., an increased urgency to feed, with only minimal effects on the rate, duration, and size of meals) in synergy with the reward system, which modulates the liking of food and motivation to work for food [80, 82]. In feeding conditions, the substantial reduction of hypothalamic and limbic forebrain 2-AG levels, suggests no implication of endocannabinoid activity in the maintenance of feeding, once the behavior is initiated [82].

Although, in mice, acute 24 h starvation led to an increase in hypothalamic 2-AG [80, 83] and AEA [84], paradoxically, a 12-day dietary restriction regimen significantly lowered 2-AG levels, and the percentage reduction was dependent on the severity of caloric restriction (60–40%) [83] (Table 1). Discrepancies in 2-AG levels in response to acute or chronic food deprivation may be related to different adaptive strategies. Acute fasting involves an upregulation of hypothalamic endocannabinoids to promote food seeking, whereas during longer periods of food scarcity, reduction in the motivation to feed (therefore attenuating 2-AG levels) ensures energy conservation and survival [82, 83].

Hypothalamic changes in endocannabinoid levels have been also implicated in the etiology of high-fat diet (HFD) preferences [85, 86], with excessive consumption of such diets leading to obesity and associated metabolic complications. The association between hypothalamic 2-AG and a HFD preference was explored using the conditioned place preference (CPP) test [85, 86], used to evaluate the rewarding effects of addictive drugs and food. The induction of place preference by HFD in the CPP test was related to a substantial increase in hypothalamic 2-AG levels to the rewarding properties of a HFD for 3–7 days after a 4-day conditioning period (animals were confined in paired boxes and a standard diet or HFD was offered for 30 min) [86]. This increase in 2-AG levels stimulates a HFD preference and activates hypothalamic astrocytes via the CB1 receptor [85]. Intriguingly, the dynamics of 2-AG levels during the HFD preference process is temporally controlled (Table 1). An initial induction phase is observed in which consumption of a HFD over 3 or 7 days leads to a transient but significant increase in hypothalamic 2-AG only after conditioning exposure to the diet. These findings agree with a similar study revealing that hypothalamic 2-AG levels increase after 10 days of HFD feeding in conditioned mice [87] (Table 1). The induction stage was followed by a maintenance phase over 14 days of HFD intake, there was a long-lasting increase in 2-AG levels, persistent activation of astrocytes, and concomitant CB1 receptor stimulation, but there was no difference in 2-AG levels in animals before and after HFD conditioning, contrasting with what has been observed after shorter periods of HFD consumption [85]. Therefore, since the temporal dynamics of hypothalamic endocannabinoid levels in response to HFD clearly differ over a few days or after longer periods of access to the diet, those dynamics could be crucial to understanding the pathophysiology of obesity and related metabolic diseases.

However, most of the studies evaluating endocannabinoid levels in the hypothalamus in diet-induced obesity (DIO) made endpoint measurements after long-term HFD exposure, once obesity was already established. In addition, results for later stages of obesity are contradictory and overlook dynamic changes in endocannabinoid levels during obesity development. In particular, hypothalamic 2-AG levels increased in mice [88] and rats [89] after long-term HFD feeding (16 and 24 weeks, respectively), but remained unchanged in mice fed a HFD for 19 weeks [58] (Table 1). In all those studies, hypothalamic AEA levels were not altered by chronic HFD exposure.

**Fig. 3:**
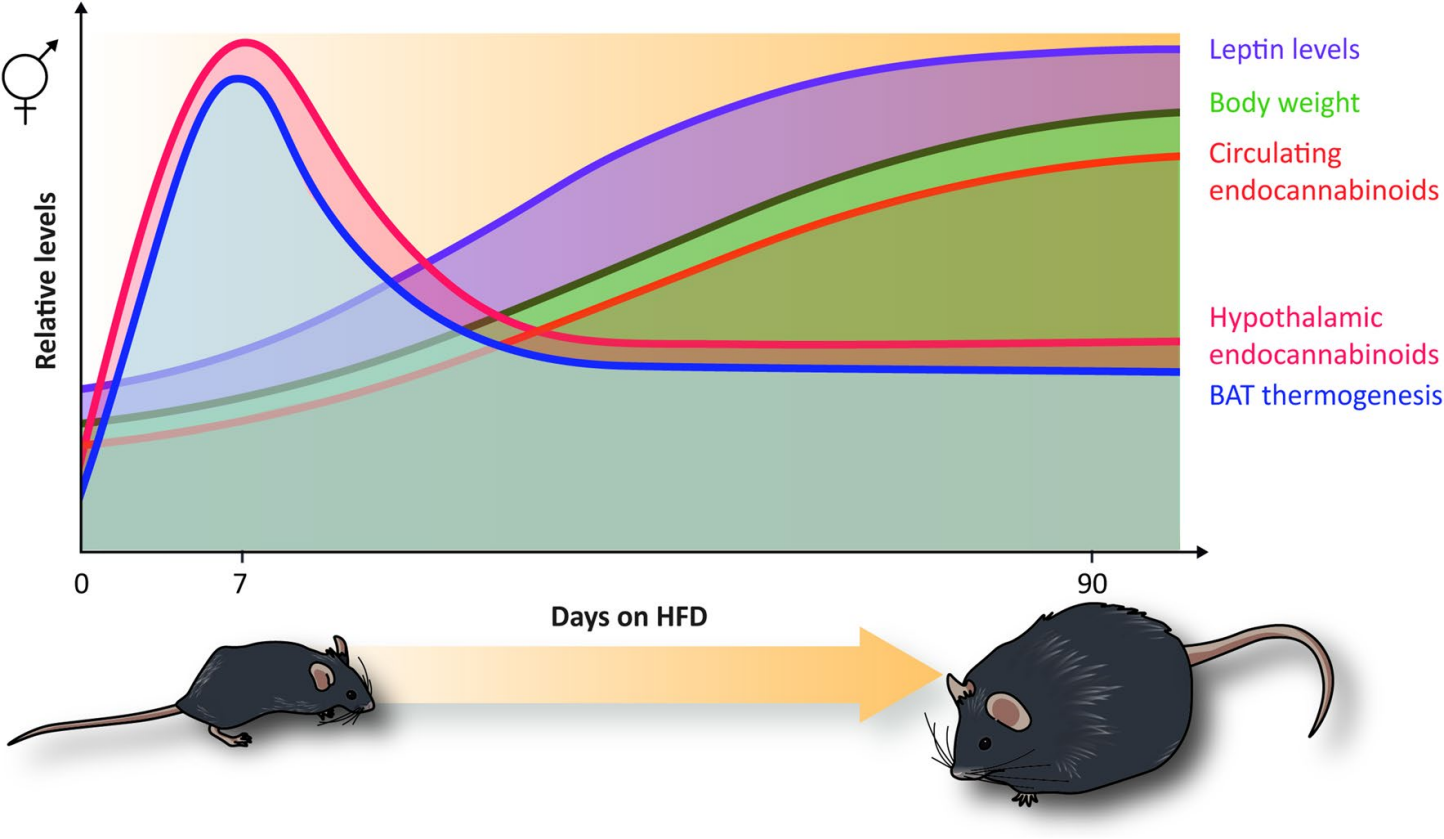
Effect of the HFD in hypothalamic and plasmatic endocannabinoids, leptin levels and BAT thermogenesis during obesity progression. During HFD-induced obesity, the progressive increase of the body weight gain (green line) positively correlates with the increase of leptinemia (purple line) and circulating endocannabinoid levels (orange line). After 7 days of HFD feeding, a transient increase of hypothalamic endocannabinoid levels was observed (red line) that positively correlated with BAT thermogenesis (blue line). This early increase of hypothalamic endocannabinoids and BAT thermogenesis gradually decreases after longer periods of HFD feeding

Considering these very few and contradictory findings, our group recently analyzed hypothalamic 2-AG and AEA fluctuations at different stages of DIO in mice and evaluated, for the first time, the association of these fluctuations to brown adipose tissue (BAT) thermogenesis activation and leptin response during obesity progression [59] (Fig. 3). A remarkable finding was that hypothalamic 2-AG and AEA levels were reduced or increased depending on stages: reduced, when DIO was already established (especially after 60 or 90 days of HFD), but increased in earlier stages (7–28 days) of HFD feeding (Table 1). A transient increase in hypothalamic 2-AG levels 4–6 times higher than basal levels was particularly evident after 7 days of HFD feeding [59] (Fig. 3); this rise was sustained by significant upregulation in hypothalamic expression of the enzymes responsible for 2-AG and AEA synthesis. Remarkably, this early and transitory increase in hypothalamic endocannabinoids positively correlated with BAT thermogenesis activation and inversely correlated with body weight gain, leptinemia, and circulating endocannabinoids (Fig. 3). Acute activation of BAT thermogenesis under different stimuli, such as leptin administration and β3-adrenoreceptor activation, also increased endocannabinoid concentrations in the hypothalamus, suggesting that this early rise in response to short-term HFD is a physiological compensatory response to BAT activation triggered by energy surfeit. The rise of endocannabinoids after short-term HFD feeding can therefore be interpreted as an early biomarker that precedes leptin resistance and peripheral obesity. Those findings also suggest the existence of one-way communication between BAT and the hypothalamus to regulate hypothalamic endocannabinoid levels. However, the exact molecular mediators involved in this cross-talk needs to be further clarified.

When analyzing the dynamics of hypothalamic endocannabinoids in response to diet, it is important to consider PUFA composition, since ARA availability is a determining factor for endocannabinoid levels in the brain [90, 91]. There are two major PUFA families: n-3 fatty acids, including EPA and DHA, and n-6 fatty acids, including the endocannabinoid precursor ARA. The amount and ratio of n-3 and n-6 PUFA intake is essential for 2-AG and AEA synthesis and, subsequently, their levels in obesity [90, 91]. Different studies have demonstrated that chronic administration high n-6 or low n-3 PUFA diets resulted in increased 2-AG levels in the whole brain and the hypothalamus [92–94]. In contrast, diets supplemented with n-3 PUFA (EPA and DHA) reduced 2-AG levels in the brain [92, 93, 95, 96]. Whole-brain 2-AG elevation after long-term administration of dietary linoleic acid (n-6, an ARA precursor) to mice induced adiposity and obesity, but adiposity and obesity was prevented by diet supplementation with EPA and DHA [92] (Table 1).

Altogether, hypothalamic endocannabinoid levels, particularly of 2-AG, are dynamically regulated in response to acute or long-term nutritional challenges, and so may play a critical role not only in appetite control but also in BAT activation, leptin-sensitive pathways, and body weight regulation. During DIO, diet duration and composition are crucial determinants of changes in endocannabinoid levels in the hypothalamus and therefore of obesity progression.

#### Last but not least: sex

Evidence increasingly points to the existence of sexual dimorphism in the different components of the ECS in rodents and humans [97–100]. It has been demonstrated that females are more sensitive to the effects of exogenous cannabinoids. Women compared to men are more susceptible cannabinoid abuse and dependence, and experience more severe withdrawal symptoms and a greater risk of relapse [97, 100]. In rodents, this higher susceptibility to cannabinoids has been observed in terms of antinociception, motor activity, and reinforcing efficacy [97, 100]. These differences have been related, on the one hand, to different pharmacokinetic responses to exogenous cannabinoids by sex [97] and, on the other hand, to lower expression in the female brain of the CB1 receptor, which, however is more efficient than in males [100].

In view of this sexual dimorphism, in recent years, research comparing males and females has been conducted on endocannabinoid dynamics. In relation to brain endocannabinoid levels, females have higher levels of the ARA-derived endocannabinoids, i.e., 2-AG and AEA, than males [101]; differences seem to be brain-region specific, and levels are also higher in the pituitary and the hypothalamus [100]. Increased AEA and 2-AG levels in female rat brains have been attributed to higher plasma availability of ARA in females [102, 103], consistent with the fact that most brain ARA is derived from the circulating pool [104]. The higher female brain levels of endocannabinoids have been also correlated with differences in the expression levels of the liver fatty acid binding protein (FABP1), a protein in charge of ARA uptake, and therefore impacting on ARA circulating levels and brain availability [101].

Martin et al. [105] found that DIO affected endocannabinoid levels differently in males and females, since ad libitum HFD (12 weeks) resulted in a smaller increase in different endocannabinoids in the brains of female compared to male mice. More recently, we evaluated hypothalamic endocannabinoid dynamics in response to HFD administration to male and female mice [59], finding that HFD increased hypothalamic 2-AG and AEA in both sexes but with significant differences between males and females; transient hypothalamic endocannabinoid increase and peak of thermogenesis activation after short-term HFD (7 days) were substantially higher in females [59] (Fig. 3). This observation is important when we consider that this transient increase in hypothalamic endocannabinoids negatively correlates with body weight and leptinemia [59] (Fig. 3). The sexual dimorphism in hypothalamic endocannabinoid levels may, for instance, contribute to explaining why obesity progression is delayed and less severe in female than in male mice. These data are also in line with previous findings on hypothalamic dimorphism in fatty acid concentration, chain length and saturation in response to HFD administration, suggesting greater protection against obesity and cardiovascular diseases in female compared to male mice [106, 107].

Sex-dependent differences in the ECS in the hypothalamus have been also reported in rat offspring in response to maternal HFD [108]. Maternal HFD promoted visceral obesity and changed CB receptor expression in the hypothalamus and BAT of rat offspring at birth, prior to obesity development. Furthermore these molecular changes occurred in a sex- and tissue-specific manner [108]; specifically, male pups presented mainly increased CB1 expression, whereas females presented increased CB2 expression [108]. These data are interesting, since sex-dependent alterations in hypothalamic ECS at birth by maternal HFD may contribute to hyperphagia, food preferences, and obesity progression later in life.

Of note, sex hormones and the female hormonal cycle can impact on brain and hypothalamic endocannabinoid levels [109, 110]. Walker et al. [109] evaluated the effect of sex and female hormonal cycle on different brain areas of the rat, finding (and confirming in subsequent studies) that female rats had more hypothalamic 2-AG than male rats, and, interestingly, that 2-AG and AEA production was affected by the estrus cycle of females [109]. Wagner et al. [110] have also extensively studied the impact of sex on CB1 signaling in hypothalamic feeding circuits. They reported that hypothalamic POMC neurons were modulated by sex hormones, as estradiol in post-synaptic POMC impaired CB1 receptor signaling at glutamatergic pre-synaptic terminals led to a reduction in food intake, whereas testosterone had the opposite effect [111, 112]. More recently, the same authors reported that SF1 neurons of the VMH furnish sexually differentiated endocannabinoid- and diet-sensitive glutamatergic inputs to ARC/POMC neurons that would suggest a basis for sex differences in cannabinoid regulation for energy homeostasis [113, 114]. For electrophysiological recordings in hypothalamic slices from intact and castrated guinea pigs, and in vitro optogenetic experiments in intact male and ovariectomized female SF1-Cre mice, the following specific findings were reported: (1) testosterone increased endocannabinoid tone at SF1/POMC synapses by increasing intracellular calcium; (2) endocannabinoid-mediated retrograde inhibition of glutamatergic input at SF1/POMC synapses was sexually differentiated and negatively regulated by estradiol; and (3) HFD feeding had a substantial impact on sex differences in endocannabinoid-induced regulation of energy homeostasis.

Thus, considering the evidence available on sexual dimorphism of the ECS in the hypothalamus and the importance of sexual hormones in energy homeostasis, in order to properly analyze the impact of gender on metabolic parameters we believe it is important to perform phenotype studies of different ECS receptors and enzymes in both sexes of transgenic animals. At the same time, further exploration of the molecular underpinning of sex hormonal action on hypothalamic endocannabinoid levels and signaling may help understand the mechanisms defining sexual dimorphism in obesity.

### Implications of modulating hypothalamic CB receptors in obesity

As discussed in the previous section, hypothalamic endocannabinoids levels are affected by hormones and nutritional stimuli that can modify CB1 action in the hypothalamus. The first study using a genetic rodent model with whole-body deletion of the CB1 receptor (CB1-KO) demonstrated that this receptor is a key component in the development of DIO; moreover, together with its endogenous ligands, CB1 is implicated not only in feeding control but also in regulating peripheral metabolism [12, 13]. Considering the importance of the hypothalamus for these processes, numerous studies have described the role CB1 in different hypothalamic nuclei and cells plays in energy homeostasis under physiological conditions and in obesity [115].

#### Hypothalamic CB1 receptors in the regulation of food intake

The earliest evidence of the CB1 receptor’s contribution to the hypothalamic control of food intake came from studies demonstrating that acute administration of AEA and tetrahydrocannabinol (THC) in the VMH or PVN induced food intake in pre-satiated rats, whereas blocking the CB1 receptor attenuated hyperphagia, thereby implying that (endo)cannabinoid action on food intake required activation of CB1 [116–118]. The CB1 receptor is co-expressed with hypothalamic neuropeptides known to modulate food intake (MCH, corticotropin releasing hormone (CRH), cocaine-amphetamine-regulated transcript (CART), and prepro-orexin), with whole-body CB1 deficient mice presenting altered expression of those genes [12]. In addition, it was observed that the anorectic action of the selective CB1 receptor antagonist rimonabant was mediated by neurons located in the dorsomedial nucleus of the hypothalamus (DMH), PVN, LH, and the ARC [119, 120].

As mentioned above, the ECS also regulates food intake by acting in extra-hypothalamic areas involved in modulating reward; for instance, direct administration of endocannabinoids in the NAc increases food intake in a CB1-dependent manner [80, 121]. Moreover, AEA microinfusions into the NAc shell increased the ‘liking’ and the intake of a sucrose solution in rats, an effect that was blocked using a CB1 receptor antagonist [122, 123]. The fact that activation of the ECS drives ‘liking’ and ‘wanting’ of palatable food further supports its implication in the development of obesity [124].

The ECS regulation of food intake not only involves different brain areas, but is also neuronal-type dependent. Intraperitoneal administration in fasted mice of low doses of THC promotes food intake whereas high doses induce hypophagia [125]. Using genetic models lacking CB1 receptor only in cortical glutamatergic or in forebrain GABAergic neurons, it was demonstrated that low doses of THC induced hyperphagia depending on CB1 receptor activation in the glutamatergic terminals, whereas high doses of THC activated the CB1 receptor on GABAergic neurons that lead to hypophagia [126]. Within the ARC, the activation of POMC neurons is thought to classically induce satiety; however, a number of studies have demonstrated that POMC neurons are molecularly and functionally heterogeneous [64]. POMC neurons are glutamatergic, GABAergic, or both, and the CB1 receptor in POMC-GABAergic neurons can modulate the hyperphagia induced by a cellular negative energy state [84].

#### Hypothalamic CB1 receptors in the control of energy metabolism

The use of genetic models of CB1 deletion in specific neuronal types has significantly contributed to understanding the role of the ECS in different hypothalamic nuclei. Using mice with CB1 receptor deletion in the forebrain (CaMK-CB1-KO) [127] and mice with a 60% reduction in CB1 receptor expression in the hypothalamus [60], it has been shown that lack of the CB1 receptor in the hypothalamus and sympathetic neurons provides resistance to DIO; when fed a HFD, those mice showed decreased body weight without changes in food intake and increased energy expenditure. Accordingly, CB1 receptor deletion in Sim-1 neurons (Sim1-CB1-KO), which account for the majority of glutamatergic PVN neurons, protected mice from weight gain in DIO thanks to increased BAT thermogenesis [128]. These results indicate that blunting CB1 receptor expression in the hypothalamus drives resistance to obesity by increasing energy expenditure and lowering adiposity. Finally, within the VMH, SF1-CB1-KO fed a chow diet displayed a mildly lean phenotype associated with increased sympathetic nervous system (SNS) activity, increased insulin and glucose sensitivity, as well as increased metabolic sensitivity to the action of leptin. However, when challenged with a HFD, SF1-CB1-KO mice displayed increased body weight gain and adiposity due to hyperphagia [66]. Therefore, CB1 specifically in VMH may represent a key player in metabolic flexibility and adaptation to different nutritional challenges.

#### The CB1 receptor in the intersection of hypothalamus-periphery cross-talk

**Fig. 4:**
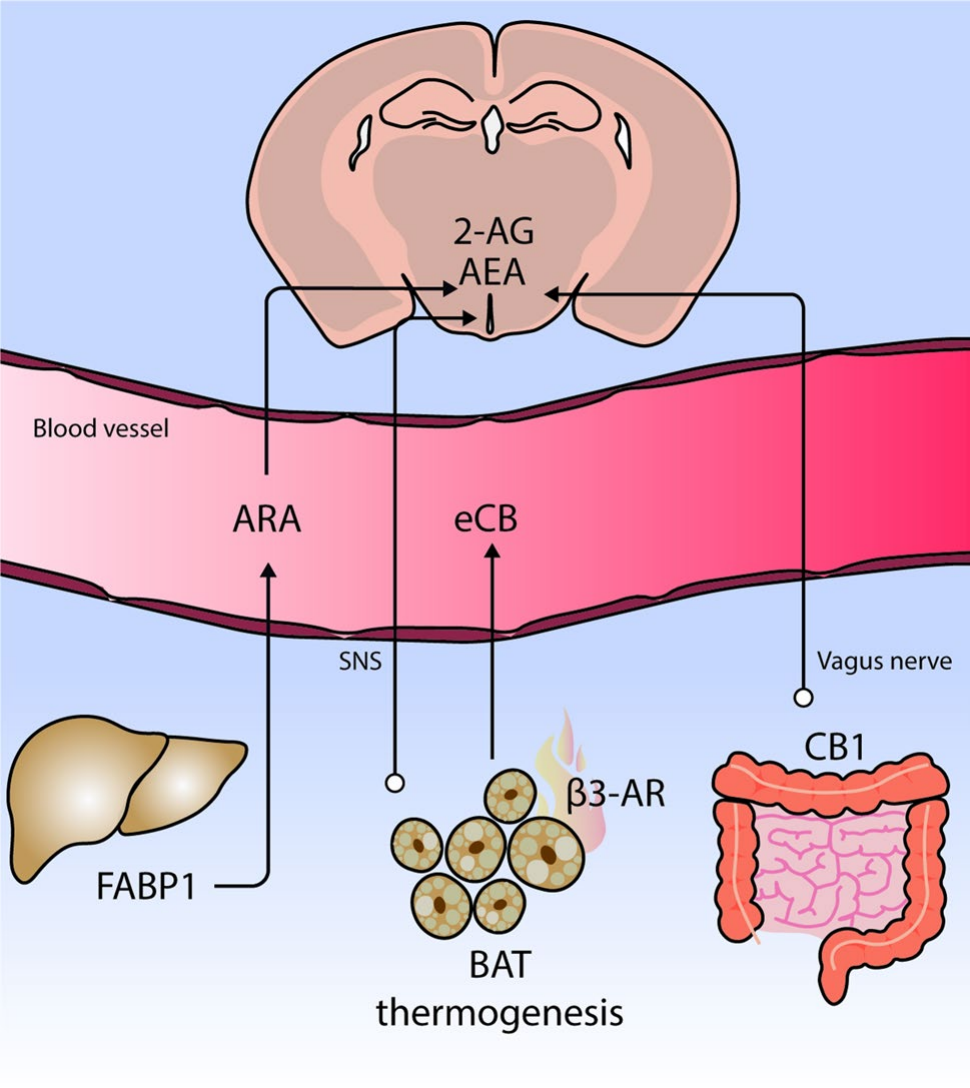
The endocannabinoid system in the intersection of hypothalamus-periphery cross-talk. Brain levels of 2-AG and AEA have been attributed to the higher plasma availability of arachidonic acid (ARA). The circulating pool of ARA is determined in part by FABP1 expression in the liver. Hypothalamic endocannabinoid (eCB) levels are modulated by brown adipose tissue (BAT) thermogenesis through the SNS tone, and BAT could be also acting as a source of circulating eCB. It has also been reported that the effect of dietary fat on gut induces a positive feed-back to the brain to modulate food intake. This response is dependent on CB1 receptors

Although numerous studies have focused on how the central ECS orchestrates peripheral metabolic responses, both peripheral CB receptor and endocannabinoid levels mediate local responses whose functional dysregulation is associated with obesity, such as BAT thermogenesis, lipid metabolism, leptin and insulin resistance, immune response, gastrointestinal tract functions, and microbiota [14]. In fact, in view of the disappointing psychiatric side effects of rimonabant, researchers have been concentrating their efforts on developing peripherally restricted CB1 antagonists for the treatment of obesity [129]. However, peripheral organs constantly and dynamically communicate with the brain to guarantee adequate control of energy balance and few investigations have focused on how the peripheral ECS may provide feedback to the hypothalamus and modulate its functions. Early evidence that the peripheral ECS mediates central responses come from the observation that different CB1 agonists and antagonists only modified food intake when injected peripherally [130]. Moreover, their effect was cancelled when capsaicin-sensitive sensory terminals innervating the gut were blocked. Indeed, exposure to fat stimulates endocannabinoid mobilization in the gut that, through the CB1 receptor, induces positive feedback driving more fat intake [131]. Rimonabant-induced hypophagia does not directly rely on targeting the CB1 receptor on brain neurons but rather on CB1-mediated SNS activity (particularly in the gastrointestinal tract), which, in turn, modulates afferent vagal fibers projecting to the nucleus of the solitary tract (NTS) [132], an area known to communicate with hypothalamic circuits to control food intake [133]. Therefore, this periphery-to-brain signaling, modulated by the ECS, represents an important mechanism implicated in the regulation of feeding [134] (Fig. 4). This mechanism may be relevant for the development of new peripherally-restricted CB1 antagonists to treat obesity. One such antagonist, JD5037, induces hypophagia by restoring hypothalamic leptin sensitivity, with the study authors suggesting that JD5037 might increase SNS activity that suppresses leptin synthesis in the adipocytes, therefore decreasing circulating leptin levels [135].

Apart from the gut, other peripheral organs send information to the hypothalamus through the ECS. As mentioned earlier, short-term HFD feeding increases endocannabinoid levels in the hypothalamus. Interestingly, icv injection of 2-AG and AEA did not promote BAT thermogenesis, whereas intraperitoneal administration of a peripheral β3-adrenoreceptor agonist, which activates BAT thermogenesis, induced a significant increase in hypothalamic endocannabinoid; this would suggest a directional link from BAT thermogenesis to hypothalamic endocannabinoids [59]. The increase in hypothalamic endocannabinoids could therefore probably be an effect mediated by increased SNS tone and BAT thermogenesis in response to diet [59] (Fig. 4). In line with those findings, a recent study has pointed to adipose tissue as a potential source of circulating endocannabinoids in obesity after HFD feeding [136], suggesting a possible connection between BAT activity and endocannabinoid levels in the hypothalamus.

Finally, Martin et al. [101, 105] demonstrated a link between fatty-acid metabolism in the liver and brain endocannabinoid levels. Mice deficient in liver FABP1, which facilitates hepatic clearance of fatty acids, showed decreased brain and plasma endocannabinoid levels compared to wild-type mice [101, 105] (Fig. 4). FABP1 modulates circulating and brain levels of ARA, a precursor of endocannabinoids, and this could explain the higher levels of 2-AG and AEA in the brain [101, 105] (Fig. 4).

Those results suggest that both the vagus nerve and SNS-dependent activation through CB1 and plasmatic endocannabinoid levels underlie the interplay between the brain and peripheral organs in ensuring an appropriate energy balance.

#### The hypothalamic CB2 receptor in the control of food intake and energy metabolism

After clinical failure of rimonabant, CB2 became a possible therapeutic target since it could avoid CB1-mediated changes on mood [34]. However, only a few studies have attempted to elucidate the role of CB2 in energy balance. The CB2 receptor has been related to food intake regulation, while the *Cnr2* polymorphism has been related to eating disorders in humans [137]. In contrast to what has been found for the CB1, CB2 receptor activation exerts a protective role against obesity. For instance, in fasted rodents, blocking CB2 receptor via icv or intraperitoneal injection using the antagonist AM630 increased food intake, while high doses of the CB2 receptor agonist JWH015 reduced food intake [34, 138, 139]. However, those results became controversial when mice treated with the CB2 antagonist SR144528 showed no modifications in food intake [35].

Since CB2 regulates immune response, the earliest study relating CB2 and obesity disorders demonstrated that CB2 activation using the agonist JWH-133 in HFD-fed mice potentiated obesity-associated inflammation, insulin resistance, and non-alcoholic fatty acid disease [140], while treatment of WT mice with the CB2 antagonist SR 144,528 resulted in improved insulin sensitivity [35]. Global deletion of the CB2 receptor in mice (CB2-KO) led to resistance to DIO, accompanied by reduced adipose and liver inflammation [35, 140]. However, CB2 receptor deficiency in aged mice induced hyperphagia associated with age-dependent obesity, and fat and liver inflammation, with no changes in insulin sensitivity [35, 36]. In contrast, overexpression of CB2 in the brain resulted reduced food intake and leaner mice, affected, however, by hyperglicemia [37]. Moreover, DIO mice chronically treated with a CB2 receptor agonist resulted in an obesity-resistant phenotype due to an improved immune response and reduced food intake [34].

Those findings demonstrate a differential role for CB2 in age-induced obesity and DIO, suggesting that CB2 is involved in food intake, glucose metabolism, and obesity-associated inflammatory processes, although in a contradictory manner. Further studies are needed to elucidate the exact role played by CB2 in the hypothalamus and in its different neuronal populations.

### New therapeutic targets beyond CB receptors

Proposed as a possible therapeutic approach to the treatment of obesity and related metabolic disorders is manipulation of endocannabinoid metabolism by targeting the enzymatic machinery to reduce endocannabinoid levels, and thus decrease food intake and increase energy expenditure [141]. However, certain issues need to be addressed, such as the lack of specificity derived from modifying enzymatic activity. Indeed, no enzyme is selective for 2-AG or AEA, and activation or deactivation of enzymes could influence the metabolism of signaling molecules others than endocannabinoids.

As mentioned above, 2-AG metabolism is mainly regulated by the synthesizing enzymes DAGLα/β and the degrading enzymes MAGL, ABHD6, and ABHD12. Several approaches to the treatment of obesity have been developed to decrease 2-AG levels by modifying this enzymatic machinery [49]. In particular, targeting DAGL represents a promising therapeutic strategy to regulate 2-AG levels and obesity development. Gao et al. [142] developed the first mouse lines with targeted disruption of DAGLα (DAGLα-KO) and DAGLβ (DAGLβ-KO). The global deletion of these enzymes resulted in a significant decrease in brain and hepatic 2-AG levels, although brain levels were much lower in DAGLα-KO than in DAGLβ-KO mice. Interestingly, AEA levels also decreased in the brains of DAGLα-KO mice. Since 2-AG, ARA, and AEA are substrates or products of the same or related enzymes, it is not surprising that major changes in 2-AG and ARA levels impact AEA levels. The reduction in brain endocannabinoids in DAGLα-KO mice was accompanied by lower body weight gain and food intake than in their wild-type littermates when mice were fed either chow [142, 143] or HFD [143]. While DAGLβ-KO mice did not show changes in body weight, DAGLβ disruption drove pro-inflammatory responses in macrophages and microglia [144, 145].

Therefore, the development of selective DAGLα inhibitors is of interest in reducing endocannabinoid tone in obesity, a statement supported by pharmacological manipulation of DAGLα in DIO models [87]. The fact that DAGLα inhibitors only counteract stimulated biosynthesis of 2-AG is an advantage, since the basaline levels will remain unaffected. Those compounds would thus act only when and where 2-AG biosynthesis is pathologically altered [87]. Inhibition of DAGLα by intraperitoneal administration of the compound O-7460 decreased food intake and body weight in mice fed a HFD in a dose-dependent manner [87]. As expected, this effect correlated with a reduction in 2-AG levels in both the hypothalamus and liver, which were increased after HFD feeding. Those findings suggest that HFD-fed mice developed a stronger HFD preference and intake due to up-regulated 2-AG levels, since DAGLα inhibition by O-7460 reduced HFD consumption by reducing 2-AG levels. In line with those results, Próspero-García et al. [146, 147] demonstrated that injection in the LH of rats of tetrahydrolipstatin (THL), a non-selective inhibitor of DAGLα and DAGLβ, decreased food intake by blunting 2-AG synthesis*.*

Enhancing the activity of endocannabinoid degrading enzymes is an alternative strategy to counteracting increased endocannabinoid tone in obesity. As mentioned above, there are three well-known 2-AG degrading enzymes that regulate specific 2-AG pools. MAGL, the main degrading enzyme, is localized in presynaptic neuronal terminals and accounts for 85% of 2-AG degradation, while ABHD6 from postsynaptic spines is responsible for 4% [47, 148–151], and ABHD12 contributes to 9% of total 2-AG hydrolytic activity [47].

Regarding MAGL, the use of transgenic animals with MAGL overexpression in different tissues shows that the effects of this enzyme on energy homeostasis are dependent on the site of expression. When overexpressed in the small intestine, MAGL facilitates diet lipid assimilation and, after HFD feeding, promotes obesity and liver steatosis, hyperphagia, and decreased energy expenditure [152]. By contrast, when overexpressed in the forebrain, HFD-fed mice show resistance to DIO, probably via an enhanced thermogenic response [153]; in that study, transgenic mice compared to WT mice showed a 50% decrease in forebrain 2-AG levels and hypersensitivity to β_3_-adrenergic stimulated thermogenesis. Surprisingly, global MAGL-KO mice showed elevated 2-AG levels but normal food intake, lipid storage, and energy expenditure [154], probably due to desensitization of brain CB1 receptors by long-term elevation of 2-AG levels [154–156]. When fed a HFD, MAGL-KO mice showed decreased body weight gain but insulin resistance. Taken together, it seems that the metabolic effects of MAGL deficiency cannot be explained only by hyperactivation of the ECS [157].

Concerning other 2-AG degrading enzymes, ABHD6, the newest member of the ECS, has been demonstrated to play a key role in the central control of energy homeostasis [158]. Even though it only deactivates a small portion of total 2-AG, it is relevant since it controls 2-AG levels at the site of biosynthesis [47, 150]. Fissette et al. [159] reported that ABHD6 in the VMH is a mediator of metabolic flexibility through regulation of 2-AG levels. Deletion of ABHD6 in neurons of the VMH by a viral-mediated knockdown approach resulted in increased 2-AG levels in this hypothalamic nucleus in mice. Importantly, those mice failed to physiologically adapt to key metabolic challenges, showing impaired feeding in response to fasting, reduced adaptive thermogenesis during cold exposure, increased susceptibility to DIO, and resistance to diet-induced weight loss when exposed to dieting (transition from a HFD to a low-fat diet). Therefore, through endocannabinoid signaling, ABHD6 in VMH plays a key role in regulating whole-body energy balance. So far, our group has demonstrated that ABHD6 hydrolase activity is specifically regulated by nutritional status in the hypothalamus and is activated on fasting as compared to the fed state [160]. In that study, we also identified carnitine palmitoyl-transferase 1C (CPT1C), an energy sensor in the hypothalamus [161, 162], as the first negative regulator of ABHD6 activity [160]. However, the role of CPT1C regulation of ABHD6 activity in whole-body energy metabolism needs further investigation.

In contrast to the function of ABHD6 in the VMH, whole-body or peripheral ABHD6 loss-of-function is protective against DIO, insulin resistance, and hepatic steatosis; this is partly explained by enhanced energy expenditure, BAT activation, and WAT browning [163–166]. Specifically, the first study that targeted ABHD6 in peripheral tissues through antisense oligonucleotides demonstrated that ABHD6 is involved in metabolic disorders induced by HFD feeding [163]. Zhao et al. [164–166] further studied the role of ABHD6 in obesity and diabetes, finding that ABHD6 is a negative modulator of insulin secretion, given that its global suppression promoted glucose-stimulated insulin secretion in β-cells [164, 165]. Moreover, the same authors demonstrated that ABHD6 suppression protected mice from DIO by enhancing both energy expenditure and WAT browning via PPARα and PPARγ activation. Corroborating this, systematic administration of the ABHD6 inhibitor WWL70 also protected mice from DIO and insulin resistance and enhanced WAT browning, as observed in ABHD6-KO mice [166]. Therefore, both studies have proposed ABHD6 as a potential therapeutic target for obesity and type-2 diabetes. However, it has been suggested that peripheral benefits of ABHD6 against complications associated with DIO and metabolic syndrome are mediated via 1-MAG signaling rather than 2-AG [167].

Most of the studies mentioned above have focused on 2-AG metabolism, but several approaches have also been developed to manipulate enzymes regulating the synthesis and degradation of AEA. Regarding the synthesis of AEA, although whole-body NAPE-PLD KO mice developed by different research groups display controversial brain lipid profiles, they did not exhibit any peculiar phenotype compared to WT mice [168–170]. The lack of phenotype suggests that compensatory mechanisms are activated when NAPE-PLD is deficient in early development stages. To overcome this problem, Cani’s Lab generated mice models in which NAPE-PLD was inactivated in specific organs (liver and adipose tissue) from adult mice, using the Cre-lox system, finding that inducible NAPE-PLD hepatocyte- or adipose tissue-specific deletion in mice led to an obesogenic phenotype, particularly in response to a HFD [171, 172].

Interesting results have also been obtained in targeting FAAH, the principal AEA hydrolase. Whole-body or peripheral FAAH-KO mice are prone to weight gain and insulin resistance due to reduced energy expenditure and increased food intake [173–175]. Balsevich et al. [58] have studied the relevance of FAAH in leptin-endocannabinoid signaling regulation of feeding and body weight under basaline and DIO conditions. Using pharmacological and genetic approaches, they demonstrated that FAAH activity is required for the hypophagic effects of leptin. Specifically, leptin increases FAAH hydrolase activity and reduces AEA levels in the hypothalamus, therefore promoting the suppression of food intake. This mechanism, however, is blunted in DIO models due to leptin resistance [58].

Altogether, although great advances have been achieved in the past few years in understanding the role of ECS enzymes in obesity development, further research is needed to clarify the regulatory roles of these enzymes in obesity, and to accurately determine their real-time levels of activity depending on nutritional status.

## Conclusion

Here we have reviewed recent literature providing new evidence on the role of hypothalamic endocannabinoids in regulating energy balance and their involvement in obesity pathophysiology. At the onset of obesity, these lipids are dynamically modulated at the onset of obesity, by diet and metabolic hormones, and regulation of their levels is sexually dimorphic. This highlights the need to study the ECS as a complex system interconnecting the functions of different organs in a physiological and pathological state, for male and female rodents simultaneously.

Beyond targeting CB receptors for the treatment of obesity and other metabolic diseases, the evidence discussed here also suggests that enzymes regulating endocannabinoid synthesis and degradation may represent promising therapeutic targets. However, several caveats and limitations remain, particularly concerning the specificity of the targets. In particular, the fact that enzymes identified to date are involved in the metabolism of several types of lipid mediators, other than endocannabinoids, leaves open the possibility of altering levels of non-endocannabinoids related lipids, whose actions on behavioral and metabolic responses are still unknown. An alternative could, therefore, be to identify a specific regulator of endocannabinoid enzymes that connect cell nutritional status of the cell with a need (or lack of need) for synthetizing and/or degrading endocannabinoids, so as to fine-tune their action in the context of maintaining the energy balance. Future studies can be expected to further provide information on such molecular mediators, e.g., the recently characterized CPT1C, as these may represent a more specific target for the regulation of endocannabinoid levels in the context of obesity and other metabolic disorders.

## Data Availability

Not applicable.

## Abbreviations

1,2-DAG: 1,2 Diacylglycerol
2-AG: 2-Arachydonoyl glycerol
4-MUH: 4-Methylumbelliferyl-heptanoate
ABHD12: α/β-Hydrolase domain containing 12
ABHD4: α/β-Hydrolase domain containing 4
ABHD6: α/β-Hydrolase domain containing 6
ABPP: Activity-based protein profiling
AC: Adenylyl cyclase
AEA: Arachidonoylethanolamide or anandamide
AgRP: Agouti-related proteinARA: arachidonic acid
ARA: Arachidonic acid
ARA: Arachidonic acid
ARC: Arcuate nucleus
BAT: Brown adipose tissue
CART: Cocaine-amphetamine–regulated transcript
CB: Cannabinoid
CB1: Cannabinoid type 1 receptor
CB2: Cannabinoid type 2 receptor
CNS: Central nervous system
COX-2: Cyclooxygenase-2
CPT1C: Carnitine palmitoyltransferase 1C
CRH: Corticotropin releasing hormone
DAGL: Diacylglycerol lipase
DHA: Docosahexaenoic acids
DIO: Diet-induced obesity
DMH: Dorsomedial nucleus of the hypothalamus
ECS: Endocannabinoid system
EPA: Eicosapentaenoic acid
FAAH: Fatty acid amide hydrolase
FABP: Fatty acid-binding protein
GDE1: Glycerophosphodiesterase 1
GHS-R: Growth hormone secretagogue receptor
GLP-1: Glucagon-like peptide 1
GPCR: G protein-coupled receptors family
HAEA: Hydroxyanandamide
HETE-G: Hydroxyarachidonoyl-glycerol
HFD: High-fat diet
HSP70: Heat shock 70 kDa protein
Icv: Intracerebroventricular
LH: Lateral hypothalamus
LOX: Lipoxygenase
lyso-PI: 2-Arachidonoyl-lysophospholipid
lyso-PLC: Lyso-phospolipase C
MAG: Monoacylglycerol
MAGL: Monoacylglycerol lipase
MCH: Melanocortin-concentrating hormone
mtCB1: Mitochondrial associated CB1 receptor
NAAA: *N*-Acylethanolamide-hydrolyzing acid amidase
NAc: Nucleus accumbens
NAE: *N*-Acylethanolamide
NAPE: *N*-Arachidonoylphosphatidylethanolamine
NAPE-PLD: NAPE selective phospholipase D
NAT: *N*-Acyltransferase
NPY: Neuropeptide Y
NTS: Nucleus of the solitary tract
PI: Phosphatidylinositol
PLA1: Phospholipase A1
PLC: Phospholipase C
POMC: Pro-opiomelanocortin neurons
PPAR: Peroxisome proliferator-activated receptor
PUFA: Polyunsaturated fatty acids
PVN: Paraventricular nucleus of the hypothalamus
SCP-2: Sterol carrier protein 2
SF1: Steroidogenic factor 1
SNS: Sympathetic nervous system
THC: Tetrahydrocannabinol
THL: Tetrahydrolipstatin
TRPV1: Transient receptor potential vanilloid type 1
VMH: Ventromedial nucleus of the hypothalamus
VTA: Ventral tegmental area
WAT: White adipose tissue
